# Synthetic carbohydrate-based vaccines: challenges and opportunities

**DOI:** 10.1186/s12929-019-0591-0

**Published:** 2020-01-03

**Authors:** Ravinder Mettu, Chiang-Yun Chen, Chung-Yi Wu

**Affiliations:** 1 Genomics Research Center, Academia Sinica, No. 128 Academia Road, Section 2, Nangang District, Taipei, 11529 Taiwan; 2 Chemical Biology and Molecular Biophysics, Taiwan International Graduate Program, Academia Sinica, No. 128 Academia Road, Section 2, Nangang District, Taipei, 11529 Taiwan

**Keywords:** Carbohydrates, Immunogenicity, Antigenicity, Polysaccharides, Vaccines and Cancer

## Abstract

Glycoconjugate vaccines based on bacterial capsular polysaccharides (CPS) have been extremely successful in preventing bacterial infections. The glycan antigens for the preparation of CPS based glycoconjugate vaccines are mainly obtained from bacterial fermentation, the quality and length of glycans are always inconsistent. Such kind of situation make the CMC of glycoconjugate vaccines are difficult to well control. Thanks to the advantage of synthetic methods for carbohydrates syntheses. The well controlled glycan antigens are more easily to obtain, and them are conjugated to carrier protein to from the so-call homogeneous fully synthetic glycoconjugate vaccines. Several fully glycoconjugate vaccines are in different phases of clinical trial for bacteria or cancers. The review will introduce the recent development of fully synthetic glycoconjugate vaccine.

## Background

Carbohydrate-based vaccines have a long history, started from the isolation of capsular polysaccharide of *Streptococcus pneumonia* (*pneumococcus*) by Dochez and Avery in 1917 [1]. Then, between 1923 and 1929, Avery and Heidelberger at the Rockefeller Institute conducted a series of studies on capsular polysaccharides (CPS) of *pneumococcus* and discovered the immunogenicity of CPS [2]. In 1930, Francis and Tillett injected pure pneumococcal polysaccharides to patients and found CPS-specific antibodies in those patients [3]. Later studies by Finland and Ruegsegger furthered the development of pneumococcal capsular polysaccharide vaccines [4]. From 1942 to 1945, Heidelberger and his associates developed tetravalent vaccine, and the test in the US army air force was successful [5].

After several clinical tests of pneumococcal polysaccharides, two variants of pneumococcal vaccines containing six serotypes each were first licensed in USA in 1946 [6]. Unfortunately, those two vaccines were discontinued shortly after due to the introduction of new and extremely effective antimicrobial drugs such as penicillin, chlortetracycline, and chloramphenicol [7, 8]. From 1950 to 1970, the antibiotics dominated the vaccine markets, and most research efforts focused on finding new antibiotics rather than developing vaccines. However, the field of pneumococcal vaccine research was kept alive by the persistent efforts of Dr. Robert Austrian who was supported and motivated by the US National Institutes of Health (NIH) towards the development of possible pneumococcal polysaccharide vaccines [9]. Meanwhile, the emergence of antibiotic resistant bacteria [10] prompted the redirection of research efforts back to the vaccine development. The unremitting efforts of Dr. Robert Austrian and his colleagues led to the development of 14-valent and 23-valent pneumococcal CPS-based vaccines that were licensed in 1977 and 1983, respectively [11, 12].

Inspired by the success of pneumococcal CPS vaccines, the tetravalent (A, C, W135 and Y) meningococcal, the *Haemophilus influenza type b* (Hib) and the *Salmonella typhi* Vi CPS-based vaccine were developed and licensed between 1982 and 1994 for adults and children older than 2 years in USA [13, 14]. Although native CPS vaccines were effective in controlling the incidence of diseases for people above 2 years of age, there were some troublesome immunological disadvantages. For example, Hib CPS vaccine elicited poor immune responses in young children below 2 years of age and immune deficient peoples whom are the more prone to infections [15]. To overcome these issues, vaccine researchers had, then, focused on increasing immunogenicity of oligosaccharides.

In 1929, Avery and Goebel demonstrated that immunogenicity of a capsular polysaccharide can be enhanced by coupling to a carrier protein [16]. Unfortunately, this finding was ignored until Robbins and Schneerson used Hib CPS (poly ribosylribitol phosphate) and DT to synthesize a glycoconjugate vaccine that exhibited greater immunogenicity and efficacy in clinical trials and was the first licensed conjugate vaccine for children younger than 2 years in the USA in 1987 [17]. The success of the Hib glycoconjugate vaccines, prompted the development of monovalentmeningococcal glycoconjugate vaccines using DT or TT as a carrier proteins to provide longer immune response and higher immunity to children younger than 2 years against serogroup C. Further extensive studies produced a quadrivalent conjugate vaccine against A, C, Y and W135 serogroups that were licensed in the USA in 2005 [18].

Moreover, conjugation technology was applied to develop an effective vaccine against important serogroups of *S. pneumoniae*. Prevenar™ (PCV7), the first licensed pneumococcal glycoconjugate vaccine produced by Wyeth laboratories in 2000, is composed of seven serogroups 4, 6B, 9 V, 14, 18C, 19F and 23F and conjugated to the nontoxic mutant of diphtheria protein CRM_197_. The results of efficacy trials showed PCV7 was safer and more effective for children younger than 2 years, and infections caused by *S. pneumoniae* significantly reduced after vaccination [19]. But the increasing cases of infections caused by non-PCV7 serotypes led to the development of PCV13 glycoconjugate vaccine, which covers six more serotypes (PCV7 + 1, 3, 5, 6B, 7F and 19A) and was approved for children from 6 weeks to 71 months in the USA in 2010 [20].

Vaccination is an effective and safe strategy to prevent infections caused by pathogens. Vaccines prepared based on the concept of conjugation generally do not display any significant disadvantages. Consequently, most countries included these carbohydrate-based conjugate vaccines in their routine immunization program [21]. Following the success of antibacterial glycoconjugate vaccines, researchers further developed carbohydrate-based conjugate vaccines for viruses, protozoans, fungi and cancer. Some of the vaccines are currently in preclinical and clinical evaluation stages [22]. Whereas many reviews covered the subject of carbohydrate-based vaccines and therapeutics [23–28], here we provided the latest advancement related to synthetic carbohydrate-based vaccines against most important pathogenic bacteria, viruses and cancer.

Over the past two decades, in addition to the traditional carbohydrate synthesis, various advanced chemical and biochemical strategies including one-pot, automated and chemo-enzymatic are being constantly developed to obtain oligosaccharides of various structures quickly in large scale with high purity for the development of carbohydrate-based vaccines and drugs [29–31].

## Main text

### Construction of carbohydrate-based vaccines

#### Natural carbohydrate-based vaccines

The majority of the licensed carbohydrate-based vaccines such as *Streptococcus pneumonia*, *Neisseria meningitides, Haemophilus influenzae* type b and *Salmonella typhi* Vi belongs to this category in which the carbohydrate antigens were isolated form microbial cultures and further conjugated to the carrier protein [32]. Despite their tremendous efficacy against corresponding pathogens, several major issues are associated with in vaccine manufacture including complicated purification procedures, heterogeneous composition, presence of cell components as an impurity, uncontrollable and unreproducible protein conjugation chemistry [33]. To overcome the above issues, chemical synthesis can produce pure, homogeneous vaccines and presents a safer and more effective alternative vaccines design.

#### Synthetic carbohydrate-based vaccines

Advances in carbohydrate chemistry have made it possible to synthesize complex oligosaccharides on a large scale. Developed in Cuba, the first commercialized synthetic vaccine, Quimi-Hib®, is a *Haemophilus influenzae* type b vaccine, which is comprised of a synthetically produced antigen conjugated to the known carrier protein TT through a spacer [34]. Some bacterial glycans and cancer antigens are available in limited quantities, presenting a difficulty in clinical trials. In such cases, synthetic chemistry can save the day by producing antigens in large quantities. Compare to biologically isolated vaccines, the advantages of synthetic vaccines include well-defined antigen structure with spacer arm, homogeneity, highly reproducibility, higher purity and better safety profile [35].

#### Fully synthetic carbohydrate-based vaccines

The third type of glycoconjugate vaccine consists of not only chemically synthetic carbohydrate antigen, but also carriers of synthetic peptides. Most of the vaccines developed for cancers and viruses fall into this category [36, 37]. However, there has not been any fully synthetic vaccine commercially available. The most promising candidates are still in preclinical stage.

### Biological application and impact of carbohydrate-based vaccines

Carbohydrates are the energy sources, mediate variety of biological functions and play a key role in numerous diseases in humans and animals. Moreover, they are potential agents in the development of carbohydrate-based diagnostics, therapeutics and vaccines [24, 26]. Over the past two decades, the vaccinology has made significant progress in protection against the infections caused by bacteria and viruses. In recent days, investigations on vaccination with pathogen-derived or synthetic carbohydrate antigens do not limit to the bacteria but extended to viruses, parasites and cancers. Some of those advancements are discussed in this section.

#### Carbohydrate-based antibacterial vaccines

Carbohydrate antigens present on the cell surface of bacteria are in the form of complex glycans and often structurally unique to be differentiated from the mammalian glycans [38]. Therefore, these complex glycans became potential targets for vaccines and biomarkers. In general, long-term use or misuse of antibiotics often lead to antibiotic resistance in pathogens. While it has not yet been observed in the case of vaccines, which target the pathogens in multiple ways by inducing T-cell responses. In addition, vaccines can reduce antibiotics usage and resistance. For example, after the introduction of the PCV conjugate vaccines into the routine childhood immunization program in several countries, the invasive bacterial diseases not only controlled but also reduced antibiotic use in vaccinated populations, and in parallel the prevalence of antibiotic non-susceptible strains also decreased [39]. Therefore, vaccination is a successful strategy to overcome the evolution of resistant strains. Thus, the success of *S. pneumonia*, *N. meningitides, H. influenzae* type b glycoconjugate vaccines has prompted researchers to develop vaccines for other pathogenic bacteria such as *Klebsiella pneumonia*, *Acinetobacter baumannii*, *Clostridium difficile*, *Staphylococcus aureus* and others to fight against their antimicrobial resistance that are presently not treatable by vaccination. In the following section, we will discuss some licensed glycoconjugate vaccines and promising synthetic vaccine candidates that are currently under preclinical and clinical trials.

#### *Haemophilus influenzae* type b (Hib)

*Haemophilus influenzae*, a Gram-negative opportunistic bacterium often inhabits the nasopharyngeal region and exists either in encapsulated or unencapsulated forms. To date six encapsulated *H. influenzae* serogroups a-f with distinct polysaccharides are recognized. Among them, Hib is more virulent in nature and causes several diseases such as pneumonia, bacteremia, meningitis and otitis media in unimmunized population particularly in children younger than 5 years old [40]. In 1987 ProHibit®, a glycoconjugate vaccine of polyribosyl-ribitol-phosphate (PRP) oligosaccharide and DT, was licensed for children younger than 2 years of age in USA. Further investigations into different types of carrier proteins offered advanced glycoconjugate vaccines with superior immunogenicity and efficacy [41].

Currently, Hib glycoconjugate vaccines with different carrier proteins, including PRP-CRM_197_ (HibTiter® by Pfizer and Vaxem-Hib® by Novartis), PRP-OMP (Pedax-Hib® by Merck), and PRP-TT (ActHib® by Sanofi Pasteur and Hiberix® by GSK) are available either in single form or in combination with other vaccines. However, these vaccines exhibit inconsistency in the PRP component sizes, the linkers types and coupled carrier protein; hence, the immune responses elicited are inconsistent [15, 32]. Since 1997, most countries introduced the Hib conjugate vaccine in the national routine child immunization programs, leading to rapid disappearance of Hib diseases in vaccine-adopted countries.

**Fig. 1:**
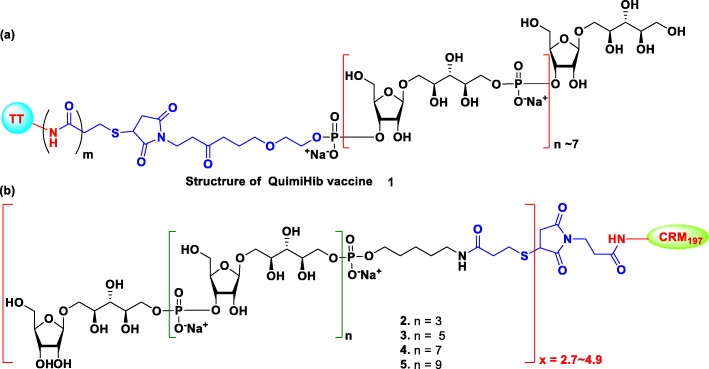
(**a**) Structure of commercially available Hib vaccine (QuimiHib). (**b**) Structure of synthetic glycoconjugates **2–5** reported by Seeberger group

To cut the cost and deal with the scarce nature of native polysaccharide Hib glycoconjugate vaccines, the Center for Genetic Engineering and Biotechnology (CIGB), Cuba, developed the first synthetic glycoconjugate Hib vaccine Quimi-Hib® **1**, which is comprised of an average seven repeating units of ribosylribitol phosphate conjugated to thiolated TT through 3-(maleimido)propanamide linker of PRP (Fig. 1a) [34]. The Quimi-Hib® vaccine **1** exhibited excellent safety profile and 99.7% protective efficacy in children. Hence, the vaccine was approved in Cuba and included in their immunization program since 2004. In order to identify the suitable length of PRP antigen for Hib vaccine design, the Seeberger group synthesized PRP oligosaccharides of various length using [2 + 2], [4 + 2], [6 + 2], and [8 + 2] iterative size elongation strategy and successfully conjugated then to CRM_197_ (Fig. 1b). Immunogenicity studies of the synthesized conjugates **2–5** on Zika rabbit model revealed that tetrameric conjugate **2** is the sufficient epitope for the new synthetic glycoconjugate Hib vaccine [42].

#### Neisseria meningitidis

*Neisseria meningitides*, often referred to as *meningococcus,* is a Gram-negative diplococcal bacterium and, causes various bacterial diseases, mainly meningococcal meningitis in young children and elderly people worldwide [43]. Among the 13 meningococcal serogroups, serogroups A, B, C, W135, X and Y are the most pathogenic strains accounting for all meningococcal infections [44]. These serogroups exhibits a geographical restriction. The serogroup A (MenA) is predominantly found in Africa and Asia, and the serogroups B (MenB), C (MenC), and Y (MenY) are most common in North America and Europe. The serogroup W135 (MenW) is found in parts of Africa and South America. Finally, serogroup X (MenX) is reported in parts of Africa [45].

To date, the development of *Neisseria meningitides* vaccines utilizes native polysaccharides, glycoconjugates and outer membrane vesicles (OMP) [46]. At present, three licensed quadrivalent meningococcal conjugate vaccines against serotypes A, C, Y and W135 are available with different brand names, Menveo® (MenA/C/W135/Y-CRM_197_, GSK), Menactra® (MenA/C/W135/Y-DT, Sanofi Pasteur), and Nimenirix® (MenA/C/W135/Y-TT, Pfizer). Although the three vaccines are different in saccharide lengths, spacer, carrier protein, and conjugation methods, they showed similar immunogenicity against the vaccine serotypes and are recommended for all age groups (2 months to 55 years). Furthermore, three licensed monovalent serogroup C conjugate vaccines and one licensed monovalent serogroup A vaccine (MenAfriVac) are available for all age groups. Two of the MenC vaccines Menjugate® (GlaxoSmithKline) and Meningtec® (Pfizer), use CRM_197_ as a carrier protein, while the third vaccine NeisVac-C® (Pfizer), use TT as its carrier protein [47].

Many attempts to develop a monovalent MenB conjugate vaccine failed because the structural similarity between the capsular polysaccharides (comprised of *α*-2,8-linked sialic acid) of MenB and components of the human neuraonal cells caused autoimmune issues in clinical tests. On the other hand, the first non-glycan-based vaccine against MenB was developed in Cuba using outer membrane protein (OMP) And the first bivalent vaccine, VA-MENGOC-BC, against MenB and C was licensed in Cuba in 1987. Later, based on reverse vaccinology, two OMP/Protein-based MenB vaccines, Bexsero (GSK, Verona, Italy) and Trumenba (Wyeth, Philadelphia, USA) were developed and approved for the age of 10 to 25 years [48].

**Fig. 2:**
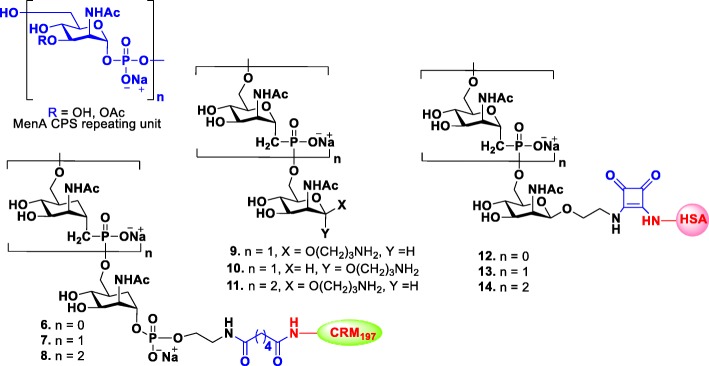
Structures of the repeating unit of MenA CPS and their synthetic 1-C-phosphono and carbocyclic analogues **6–14**

Moreover, research efforts have been devoted to the development of effective synthetic glycoconjugate vaccines for meningitis. The CPS structure of MenA is constructed by (1 → 6)-linked 2-acetamido-2-deoxy-*α*-D-mannopyranosyl phosphate repeating units with 70–80% *O*-acetylation at 3-OH (Fig. 2) [49]. The Pozsgay and Oscarson groups independently reported the synthesis of MenA CPS fragments, up to trisaccharide and cannot be further extended due to fragments instability [50, 51]. Correspondingly, the native MenA CPS also suffers from poor stability in water due to the breaking of the anomeric and phosphodiester bond by the assistance of the adjacent NAc group [52].

In order to overcome this problem, an anomeric oxygen or ring oxygen atom of pyranose with methylene group, respectively, was substituted to synthesize stable 1-C-phosphono and carbocyclic analogues of the MenA CPS repeating unit (Fig. 2) [53, 54]. Adamo and Lay recently reported the synthesis of CRM_197_ conjugated carbocyclic monomer, dimer and trimer analogous **6–8** and evaluated their immunogenicities in mice [55]. All the synthesized glycoconjugates **6–8** elicited carbasugar specific antibodies that recognized their respective structures, but only the conjugate trimer **8** was able to induce specific anti-MenA IgG antibodies with detectable in vitro bactericidal activity although in a less extent than hexamer and pentadecamer native polysaccharide CRM_197_ conjugates. Similarly, 1-C-phosphono analogues of MenA CPS **9–11** were synthesized, and their immunological properties were investigated. Competitive ELISA assays showed that all synthetic fragments with unnatural phosphonoester linkage were clearly recognized by human polyclonal anti-MenA antibodies [56]. Recent studies showed that all HAS conjugates of 1-C-phosphono analogues **12–14** were able to induce both in vitro T-cell proliferation (40% proliferation at 10^2^μM) and in vivo specific IgG production [57]. Overall, these studies suggested that chemical modifications do not prevent an immune response. Hence, carbocyclic and 1-C-phosphono analogues of MenA CPS could also serve as vaccine candidates, and its longer oligomers may induce comparable immune response to that of commercially available vaccine.

**Fig. 3:**
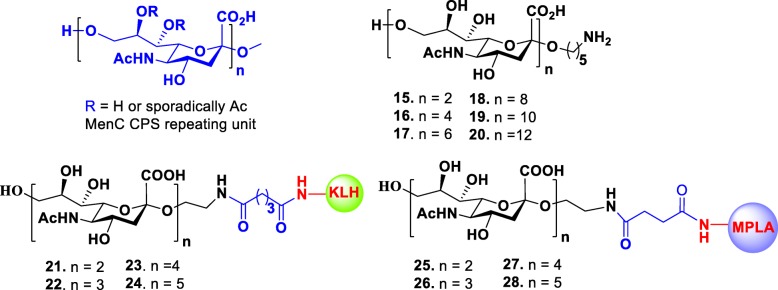
Structures of the repeating unit of MenC CPS, their synthetic oligosaccharides **15–20** and glycoconjugates **21–28**

The CPS of MenC is composed of *α*-(2,9)-polysialic acid with sporadic 7/8-*O*-acetylation (Fig. 3). Non-acetylated fragments are also immunogenic and can induce an immune response [58]. In order to develop a synthetic vaccine against meningitis, Wu and Wong group synthesized a series of non-acetylated *α*-(2,9)-oligosialic acids of various lengths ranging from dimer to dodecamer **15–20** by a convergent synthetic route [59]. Later, the Guo group adopted the same synthetic strategy to successfully synthesize *α*-(2,9)-sialic acid oligomers ranging from dimer to pentamer and conjugated them to KLH for immunological study in a mice model. They discovered that all conjugates **21–24** were immunogenic and elicited specific antibodies that only recognized the *α*-(2,9)-polysialic acid expressing *N. meningitidis* cells [60]. The same group recently reported a new type of fully synthetic vaccines **25–28** that are composed of *α*-(2,9)-oligosialic acids and monophosphoryl lipid A (MPLA), which also acts as self-adjuvant [61]. Immunological studies of these conjugates in mice revealed that they alone elicited strong immune response that was comparable to corresponding KLH-conjugates plus adjuvant. The elicited antibodies (IgG2b and IgG2c) had strong specific binding to *α*-(2,9)-oligosialic acids and polysaccharides of MenC cells. Among the tested MPLA conjugates, trimer **26** and tetramer **27** elicited highest titers of antibodies and emerged as promising vaccine candidates worthy of further investigation.

**Fig. 4:**
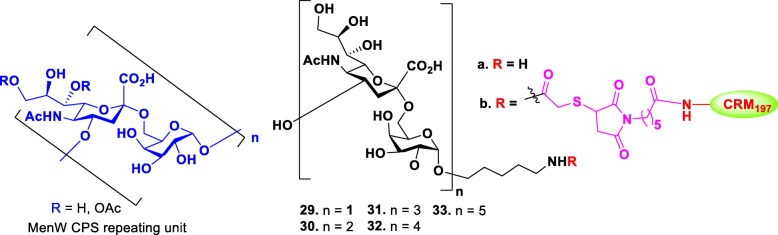
Structures of the repeating unit of MenW CPS and their synthetic glycoconjugates **29–33**

The MenW CPS consists of a glycan repeating unit of [→6)-*α*-D-Gal*p*-(1 → 4)-*α*-D-Neu*p*5Ac(7/9*O*Ac)-(2→] (Fig. 4). Wu group reported the first synthesis of MenW CPS oligosaccharides in various lengths from di- to decasaccharides **29a-33a** and determined the appropriate minimal structure for the development of synthetic vaccine [62]. Oligosaccharide chain elongation was accomplished by iterative glycosylation and deprotections using disaccharide as common donor through [2 + n] glycosylation strategy. The synthesized oligosaccharides were conjugated to CRM_197_ for immunogenicity study in a mice model. Microarray analysis and bactericidal activity assay demonstrated that immunization of vaccine candidates **30b-33b** elicited antibodies that could recognize tetra- to decasaccharides, but the vaccine candidate **29b** did not recognize disaccharide. Among the longer oligomers, the tetramer **32** elicited antibodies with the highest bactericidal effect. These results suggested that the tetrasaccharide **30** is the minimum saccharide length required to induce bactericidal antibodies.

**Fig. 5:**
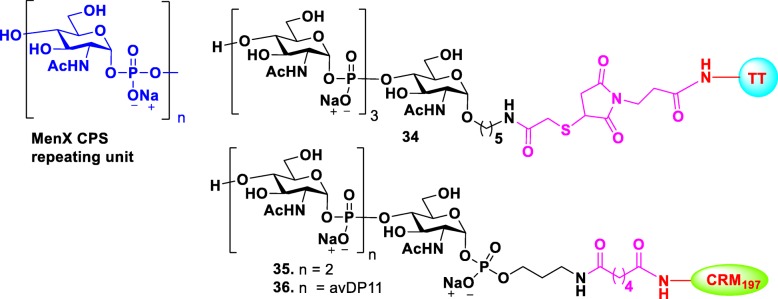
Structures of the repeating unit of MenX CPS and their glycoconjugates **34–36**

Over the past 5 years, incidence of meningitis caused by MenX has increased in the “meningitis belt” area (Sub-Saharan Africa). However, no available vaccines can prevent MenX. Recently, native CPS-based glycoconjugate vaccines of various lengths and different conjugation chemistries were demonstrated to be effective in producing high IgG antibody levels in mice, and the elicited antibodies showed effective serum bactericidal activity [63]. As an alternative for the native MenX polysaccharide, a tetramer-TT glycoconjugate [64] **34** and a trimer-CRM_197_ glycoconjugate [65] fragment **35** of MenX were synthesized (Fig. 5) and their immunological properties were tested. Although both conjugates exhibited immunological properties, they were lower than that of natural polysaccharides. However, when oligomers were longer than three repeat units, the elicited immunogenicity that was comparable to that by native polysaccharides. Recently, a longer MenX oligomer with a controlled average length was generated by enzyme-catalyzed one-pot elongation procedure [66]. The prepared oligomer was conjugated to CRM_197,_ for immunological study in a mice model. Glycoconjugate **36** elicited functional antibodies that were comparable to the antibodies from the controls immunized with MenX glycoconjugates prepared from the natural or enzymatically prepared CPS.

#### Streptococcus pneumonia

*Streptococcus pneumonia* are remarkable Gram-positive bacteria and cause life-threating diseases such as, pneumonia, meningitis, and septicemia in pediatric and elderly populations who are not protected by the pneumococcal vaccines. Based on the chemical structure of their CPS, 97 serotypes (ST) of *S. pneumoniae* were identified, of which about 20 are virulent in nature and responsible for 90% pneumococcal diseases [67]. According to the recent survey, *S. pneumoniae* caused 1,189,937 deaths (95% UI 690445–1,770,660) in people of all ages worldwide in 2016 [68].

Currently, two types of vaccines against *S. pneumoniae* are available. One is 23-vlaent native polysaccharide-based pneumococcal vaccine PPV23 (Pneumovax®23) that contains 23 purified CSPs recommended for people of and above the age of 50 years. The second type is the glycoconjugate vaccine such as PCV10 (Synflorix®) and PCV13 (Prevnar13®). Synflorix® is a 10-valent glycoconjugate that contains three different carrier proteins (PhiD, TT and DT) and approved for children from 6 weeks through 5 years. And Prevnar13® is a 13-valent glycoconjugate vaccine with CRM_197_ carrier protein and was licensed to use in infant, children and adults from 6 weeks through 65 years [69]. In addition, a 15-valent glycoconjugate vaccine developed by Merck has recently completed Phase 3 clinical trials and will soon be available in the market [70].

Although existing pneumococcal conjugate vaccines (PCVs) are highly effective in preventing pneumococcal disease in infants and children, they are not without limitation. Current PCVs do not cover all serotypes and only provide protection against serotypes included in vaccines. Specifically, PCV13 exhibited lower immune efficacy against serotypes 3, 6B, and 23F, and PCV10 against 19F at pre-booster. None of these PCVs provided enough immune protection against serotypes 1, 4 and 5 [71–73]. An alternative option to isolation is to design vaccines based on synthetic oligosaccharides providing vaccine candidate not only in pure and homogeneous form but also with lower vaccine manufacture costs.

**Fig. 6:**
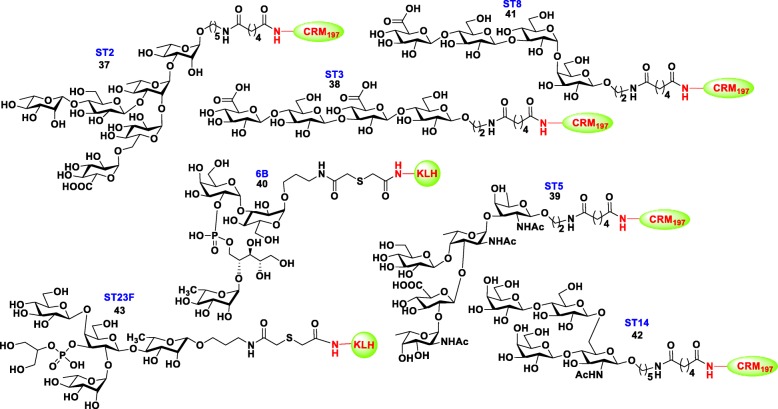
Structures of the minimal synthetic oligosaccharide-protien conjugates of *S. pneumoniae* serotypes ST2, ST3, ST5, ST8, 6B, ST14 and ST23F (**37-43**)

In the past few years, various methods have been developed to identify effective carbohydrate epitopes that can induce protective immunity in vivo that is generally required for vaccine development [74]. In the development of synthetic vaccines for *S. pneumonia,* various research groups have reported immunogenicity, antigenicity and protective effects of synthetic oligosaccharide-protein conjugates (neoglycoconjugates) of *S. pneumoniae* serotypes ST2, ST3, ST5, ST6B, ST8, ST14 and ST23F in various lengths, frameshifts, and different carrier proteins in animal models. Using ELISA and microarray, suitable minimum synthetic epitopes of all those bacteria were identified **(**Fig. 6) for the development of carbohydrate-based third-generation pneumococcal vaccines. Most of these neoglycoconjugates elicited higher titers of opsonic antibodies with prolonged memory compared to traditional conjugated vaccines in animal models [75, 76].

#### Shigella

*Shigella* are gram-negative bacteria that belongs to Enterobacteriaceae family and causes shigellosis, which is an intestinal infection that leads to severe diarrhea and abdominal cramps in humans worldwide [77]. Shigellosis is an important health problem and economic burden for developing countries. A recent study reveals that *Shigella* was the second leading pathogen that caused diarrhea and hospitalization for around 2.69 million people and 2,12,438 deaths (95% UI 136979–326,913) globally in 2016 [78].

Based on the biochemical properties, around 50 serotypes of the *Shigella* were identified and classified into four species including *S. dysenteriae* (15 serotypes), *S. flexineri* (15 serotypes), *S. boydii* (19 serotyps) and *S. sonnei* (1 serotype). Among them, *S. flexineri* and *S. dysenteriae* are more virulent in nature, whereas *S. sonnei* is generally least virulent [79].

Although various traditional vaccine strategies have been attempted for developing safe and effective *Shigella* vaccines for decades, no vaccines against *Shigella* have been licensed. Most of the vaccine candidates are in various clinical stages [80, 81]. In addition to these traditional efforts, a number of studies have attempted to use synthetic glycoconjugate to develop shigella vaccines, and some are currently under various clinical studies [82].

**Fig. 7:**
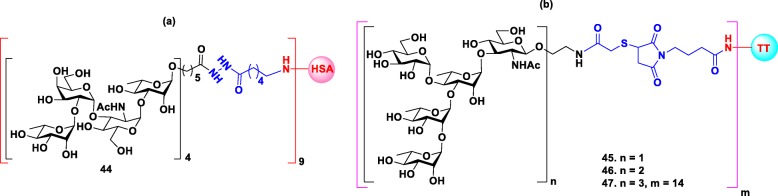
Structures of (**a**) Synthetic glycoconjugate against *Shigella dysenteriae* type- 1 **44**. (**b**) Synthetic glycoconjugates against *Shigella flexneri* 2a **45–47**

*S. dysenteriae* type 1 is a major causative pathogen of dysentery caused by the release of potent Shiga toxin. The first synthetic glycoconjugate vaccine against shigellosis was reported by Pozsgay group [83] that consisted of four repeating units of the tetrasacchride [*α*-L-Rha-(1 → 2)-*α*-D-Gal-(1 → 3)-*α*-D-GlcNac-(1 → 3)-*α*-L-Rha] O-specific polysacchride (O-SP) of the LPS of *S. dysenteriae* type 1 covalently bound to HSA through heterobifunctional spacer (Fig. 7a). The immunological studies in a mice model revealed that hexadecasaccharide conjugate **44** with an average of nine chains of saccharides per protein molecule was the most immunogenic epitope that elicited higher level of anti O-SP related IgG antibodies in mice than the isolated O-SP-HAS conjugate.

*S. flexneri serotype 2a* is the most prevalent pathogen of *S. flexneri* and responsible for endemic shigellosis among children in developing countries*.* Specifically, an important virulent factor is that *S. flexneri* expresses O-specific polysaccharides (O-antigen) as a part of LPS. The O-antigens of all *S. flexneri* except serotype 6 share a common linear tetrasaccharide repeating unit →2)-*α*-L-Rha-(1 → 2)-*α*-L-Rha-(1 → 3)-*α*-L-Rha-(1 → 3)-*β*-D-Glc*p*NAc-(1 → as a backbone [84]. Due to its structural similarity to other serotypes but with more pathogenicity, Serotype 2a is considered as a suitable target for *shigella* vaccine design. In order to develop a synthetic glycoconjugate vaccine against shigellosis*,* Mulard group synthesized monomer, dimer and trimer of the pentasaccharide repeating unit of O-antigen of *S. flexneri 2a,* and conjugated them to maleimide activated TT protein for immunological study in a mice model (Fig. 7b) [85]. And the results of the immunogenicity studies showed that when the size of the oligosaccharide increased from monomer to dimer to trimer **45–47** the IgG response also improved. Moreover, pentadecasaccharide glycoconjugate **47** induced specific and long-lasting anti O-SP 2a antibodies in mice. Further studies demonstrated that anti-OSP 2a antibodies induced by glycoconjugate **47** could protect the mice from *shigella* infection, suggesting that pentadecasaccharide is a strong candidate for vaccine development. Currently, the vaccine candidate **47** has already entered Phase II clinical trial with promising results [86].

#### *Bacillus anthracis*

Anthrax is an infection disease caused by spore forming, Gram-positive bacterium, *Bacillus anthracis* that exists in two forms, vegetative cells and spores. In adverse environments, the vegetative *B. anthracis* is able to convert into spore form (endospore), which is highly resistant to heat, radiation, pH and harsh chemicals, allowing it to persist in the soil and other environments for decades until favorable growth conditions occurs. Due to its highly pathogenic nature, mortality rates, and ease of spreading, *B. anthracis* is considered as an agent of bioterrorism [87]. The spores of *B. anthracis* can enter humans and animals by three different modes including skin lesions, inhalation and ingestion. Then, the entered spores circulate through blood stream and germinate to their vegetative form that commences rapid replications and release the toxins. This entire process takes place within a few days to few weeks, and early diagnosis and treatment are unlikely [88]. Capsular polysaccharides and anthrax toxin are the main virulence factors of *B. anthracis*. Anthrax toxin is a tripartite exotoxin composed of three proteins known as the edema factor (EF), the lethal Factor (LE) and the protective antigen (PA). Individually, these three proteins are nontoxic, but in binary combinations, particularly PA with EF and PA with LE, they produce edema toxin (ET) and lethal toxins (LT), respectively [89].

Although anthrax can be treatable by antibiotics, vaccination is the best option to prevent anthrax. So far, the first and second generation of human anthrax vaccines have been developed based on the spores and anthrax toxin. However, the vaccines have several limitations, including poor immunogenicity, tedious 5- to 6 primary vaccination doses with annual boosting, low efficacy, uncertain safety, and side effects [89, 90]. Therefore, there is a need to develop a new type of vaccines with novel formulations. In this regard, the development of well-recognized glycoconjugate vaccines is one of the major choices. The glycans present on the surface of the *B. anthracis* vegetative cell and spores provide broad opportunities for the development of new vaccines and biomarkers against anthrax [91].

**Fig. 8:**
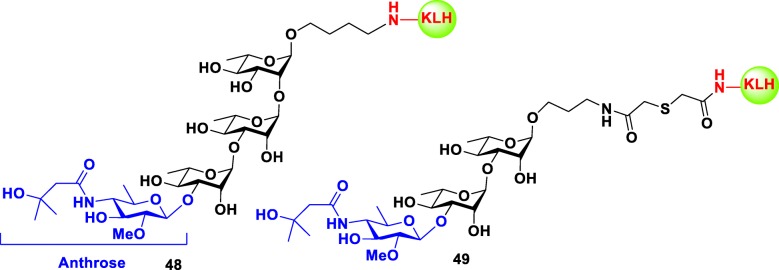
Structures of the synthetic glycoconjugates **48–49** against *Bacillus anthracis*

Many preclinical studies have focused on the tetrasaccharide expressed on the surface of *B. anthracis* exosporium. This tetrasacchride is composed of three rhamnose resides and one rare sugar known as anthrose at its nonreducing end [92]. Seeberger group was the first to demonstrate that synthetic anthrax tetrasaccharide bound to KLH protein **48** (Fig. 8) are immunogenic in mice. The resulted carbohydrate specific monoclonal IgG antibodies recognized the glycan structure of native *B. anthracis* endospores [93]. Further studies by Boon group showed that anthrose-rhamnose-rhamnose trisaccharide conjugated to KLH **49** (Fig. 8) was a sufficient fragment to bind to the antispore rabbit serum and the isovaleric acid substituent of anthrose played a crucial role in antibody recognition [94]. Later studies by various groups mainly focused on the role of anthrose residue and its structural requirements in immunogenicity and antigenicity. The results of these studies can be summarized to i. anthorse is the immunodominant feature of the tetrasaccharide; ii. isovaleric acid moiety at C-4 and methyl group at C-6 of anthorse are key antigenic elements and essential in recognition of anti-spore antibodies; iii. OMe group at C-2 is not necessary, because it is not involved in recognition of antibodies; and iv. rhamnose moiety alone (without anthrose) is not crucial to antigenicity. To date most of the glycoconjugate vaccines developed against anthrax are still in preclinical stage.

#### *Clostridium difficile*

The gram-positive, spore forming, and toxin-producing bacterium, *Clostridium difficile,* mainly causes nosocomial antibiotic-associated colitis and diarrhea in humans. Over the past 10 years, *Clostridium difficile* infections (CDI) have emerged globally. In the USA alone, the estimated CDI cases reached 606,058 and CDI-attributed deaths reached 44,572 in 2014, translating to an economic burden of $ 4 to7 billion USD annually [95]. Like *B. anthracis*, *C. difficile* can also exist as spores, which are able to survive for months in all environments without loss of viability and can transmit to people via the oral route. After ingestion, spores can survive in the stomach and subsequently reach to intestine, and patient remains disease free at this stage. When the balance of natural gut microbiota is disturbed by antibiotics treatment of other diseases, the environment favors the spores to germinate into vegetative cells that can enter into the colon and secrete two enterotoxins (TcdA and TcdB) that can severely damage the intestinal mucosa and lead to colitis and diarrhea [96]. On the other hand, *C. difficile* strains that do not produce toxins are non-pathogenic.

Although CDI can be treated by antibiotics, there is still an urgent need of *C. difficile* vaccines due to the emergence of antibiotic resistant strains, recurrent CDIs, difficulty in diagnosis and economic burden of the treatment. Over the past decade, most research effort has been focused on the development of *C. difficile* toxoids-based vaccines, which are currently in different stages of clinical trials [97]. Apart from that, carbohydrate-based vaccines are studied at preclinical level. Although *C. difficile* spores don’t express any surface gycans, the vegetative form of *C. difficile* cells do express three types of glycans (PSI, PSII, and PSIII) on the cell surface. Among them, PSII is the most abundant polysaccharide and is expressed by all *C. difficile* ribotypes and thus, represents an important target molecule for vaccine design [98].

**Fig. 9:**
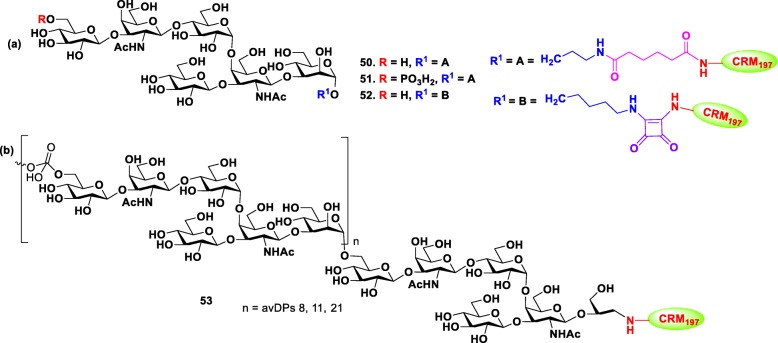
Structures of (**a**) PSII synthetic glycoconjugates **50–52** against *Clostridium difficile*. (**b**) Native PSII-CRM_197_ glycoconjugate **53**

Two groups individually investigated the synthesis, immunogenicity and antigenicity of PSII oligosaccharide of *C. difficile*. In order to study the role of phosphate group in immunogenicity, Adamo et al. first synthesized the hexasaccharide repeating unit of PSII with and without phosphate group at non-reducing end via [4 + 2] convergent approach [99]. The synthetic antigens and native PSII polysaccharide were conjugated to CRM_197_ carrier protein, respectively Fig. 9 (Hexa-CRM_197_ **50**, HexaP-CRM_197_ **51** and PSII-CRM_197_ **53**), and the glycoconjugates were used to immunize Balb/C mice. Interestingly, IgG antibodies elicited by both native PSII-CRM_197_ **53** and synthetic HexaP-CRM_197_ **51** glycoconjugates were able to recognize PSII on the surface of *C. difficile* cells. However, nonphosphorylated Hexa-CRM_197_ **50** did not induce either IgG or IgM antibodies, indicating the importance of negatively charged phosphate group for immunogenicity. Concurrently, Seeberger group completed another study, in which the mice were immunized with a conjugate **52** composed of the synthetic nonphosphorylated PSII hexasaccharide that bound to CRM_197_ carrier protein through squaric acid [100]. The neoglycoconjugate **52** was immunogenic in mice and produced carbohydrate specific antibodies that specifically interacted with the synthesized glycan hapten. These results suggested that PSII hexasaccharide single repeating unit with charged phosphate group is the sufficient potential epitope for vaccine design against *C. difficile*. In addition, the immunogenicity of PSI and PSIII oligosaccharides were also studied using mice and rabbit models.

#### Brucella

*Brucella* species are non-spore-forming, gram-negative coccobacilli that causes Brucellosis in humans and animals such as cattle, goats, camels, sheep, deer, swine and dogs worldwide. Among the 10 species within the genus *Brucella B. melitensis*, *B. abortus*, *B. suis* and *B. canis* are the main pathogenic species in both animals and humans [101]. Brucellosis is an endemic and mostly transmitted to humans by direct contact with the infected animals or consumption of their raw milk and meat products [102]. The emergence of human brucellosis is a serious problem and effects the economy in developing countries such as India, China, Brazil and some of the African countries. The available diagnostic tools of *Brucella* are inadequate, expensive and time consuming. Moreover, the available live vaccines are limited to ruminants, and there is no vaccine for humans [103]. Furthermore, treatment of human brucellosis requires long and costly antibiotic therapy. Therefore, there is an urgent need to develop a superior diagnostic tools and vaccines against *Brucella* [104].

**Fig. 10:**
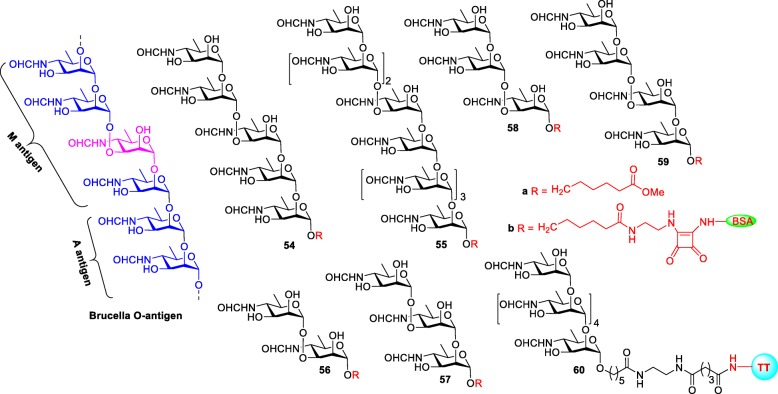
Structures of the *Brucella* O-antigen and their synthetic oligosaccharides **54a-59a** and glycoconjugates ** 54b-59b** and **60**

The O-antigen or O-polysaccharide (OPS) domain of LPS of *Brucella* is composed of a homopolysaccharide rare sugar 4,6-dideoxy-4-formamido-*α*-D-mannopyranose (Rha4NFo) that exists in two sequences, resulting in two types of antigens known as A and M antigens (Fig. 10). The A antigen consists a longer inner sequence of *α*-1,2-linked D-Rha4NFo residues and is capped by the M type antigen, which contains one *α*-1,3-linked D-Rha4NFo for every four *α*-1,2-linked D-Rha4NFo resides [105]. Both A and M antigens are virulent in nature, and studies showed all of the investigated *Brucella* strains have 2 to 21% of M character linkages except for *B. suis biovar* 2, which only has A type antigen [106].

In 2013, the Bundle group synthesized pentasacchride **54a** and nonasacchride **55a** of O-antigen and studies their antigenicity [107]. The nonasacchride **55a** was designed to have A and M epitopes, whereas pentasacchride **54a** had mostly M type. After the conjugation with BSA, both conjugates **54b** and **55b** were coated on ELISA plates, to be tested against two monoclonal antibodies (mAbs) YsT9–1 and Bm10, specifically for the *Brucella* A and M antigens, respectively. Interestingly, nonasacchride antigen **55b** bound to A- and M-specific mAbs with equivalent avidity, whereas the pentasaccharide antigen **54b** preferentially bound to M-specific mAbs, as expected. This discrimination between M and A antibodies by pentasaccharide conjugate might improve by decreasing the number of 1,2-linked α-D-Rha4NFo residues in the molecule.

To study this possibility, a series of M-type oligosaccharides from di- to tetrasacchrides **56a-59a** was synthesized and subsequently conjugated to BSA to identify the smallest and largest M epitopes [108]. Surprisingly, both di- and tetrasaccharide-BSA conjugates **56b** and **59**b (M-type) were able to detect antibodies in the sera of humans and animals infected with *B. suis* and *B. abortus*, despite of having A-dominate LPS in their cell wall. Moreover, the same conjugates also showed strong binding avidity to M-specific mAbs and weak to negligible binding to A-specific mAbs. Furthermore, the anti A-antibodies elicited exclusively by *α*-1,2-linked hexasaccharide-TT conjugate **60**, bind well to the M type disaccharide and tetrasaccharide antigens **56b** and **59b** [104]. These results suggested that disaccharide antigen **56** is the simplest structure that can detect antibodies in the sera of *Brucella* infected animals and humans and would be a promising biomarker for the detection of *Brucella*.

### Carbohydrate-based anti-cancer vaccines

**Fig. 11:**
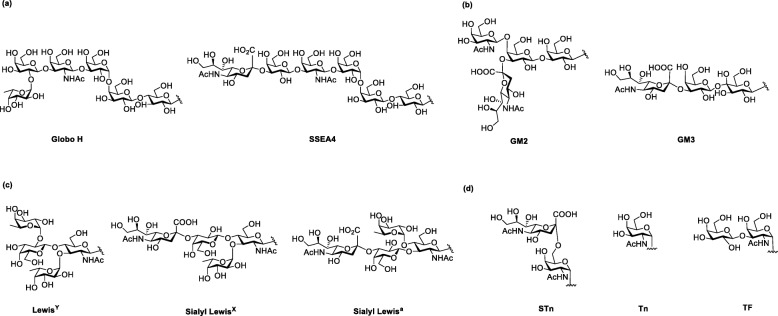
Structure of representive TACAs: (**a**) Globo series; (**b**) Gangliosides; (**c**) Blood group; (**d**) Mucin attached glycan

Cancer is a type of disease with immortalized cell growth and metastasis to other tissues of human body. Vaccines for cancer treatment are classified into prevention vaccines, which prevent virus infection (e.g., HPV vaccine against human papillomavirus and Hepatitis B vaccine against hepatitis B virus), and therapeutic vaccines, which are immunotherapy that train and activate immune system in human body to eliminate cancer cells (e.g., Provenge® against prostate cancer). Recently, immunotherapy is gaining popularity in cancer treatment due to its low side effects and high specificity [109]. Most of the immunotherapies target on the surface protein such as PD-L1 of the cancer cell. In addition, tumor-associated carbohydrate antigens (TACAs), which are abundant on the surface of different types of cancer cells, are highly associated with tumor progression and therefore potential candidates for cancer immunotherapy [110, 111]. TACAs are classified into four groups (Fig. 11): (1) The Globo series including Globo H, SSEA4 and SSEA3 (GB5) which are glycolipids and overexpress in breast, prostate, lung, ovary and colon cancer cells; (2) the gangliosides including GD2, GD3, GM2, GM3 and fucosyl GM1 which overexpress on melanoma, neuroblastoma, sarcoma and B-cell lymphoma; (3) the blood group including Lewis^X^, Lewis^Y^, sialyl Lewis^X^, and sialyl Lewis^a^ which are also gangliosides and overexpress on breast, prostate, lung colon and ovary cancer cells; (4) the glycoprotein including Thomsennouveau (Tn), Thomsen−Friendreich (TF), and sialyl-Tn (STn) which attach at the serine/threonine on the mucin and overexpress in epithelial cancer cells (breast, ovary and prostate) [112–119]. Previous clinical experiences showed increasing survival rate in patients who were passively administrated antibodies recognizing carbohydrate or generated appropriate amount of antibodies after immunization with carbohydrate-based vaccine [120, 121]. Thus, TACAs are demonstrated to be ideal targets for cancer vaccine development.

#### TACAs with protein carrier

TACAs are poorly immunogenic and T-cell independent, similar to bacterial polysaccharides as mentioned earlier. Therefore, many studies covalently conjugated TACAs to carrier proteins such as BSA, KLH, DT, TT, OVA, and MUC1 peptides to induce T-cell mediated immune response [28]. Interestingly, the same TACA with different carrier proteins resulted in different immune response against TACA. For example, Helling et al. conjugated ganglioside antigen GD3 to different carrier proteins BSA, KLH, OMP, multiple antigenic peptide (MAP) and polylysine through reductive amination [122]. After immunization the conjugates to mice, the strongest IgG antibody titer was found in mice with GD3-KLH and QS-21 immunization. Similarly, Danishefsky and Livingston’ group synthesized several Tn (consist of monosaccharide GalNAc) constructs: Tn monosaccharide, Tn-threonine trimer cluster, and Tn partially or fully glycosylated MUC1 cluster and conjugated them individually to KLH or BSA carrier protein through m-malemidobenzoyl- N-hydroxysuccinimide ester [123]. They found that Tn-KLH induced stronger IgG titer than Tn-BSA. As a part of cancer vaccine development, our group has synthesized Globo H vaccines with KLH, DT, TT, and BSA carrier proteins and immunized them in a mice model with different adjuvant. We found that Globo H-DT with C34 adjuvant induced the strongest IgG antibodies that specifically recognized Globo series antigens (Globo H, SSEA4 and SSEA3) [124].

To conjugate TACAs to the carrier protein, the reducing end of TACA is installed with spacers including *p*-nitrophenyl, maleimide, aldehyde containing groups, which then conjugated to carrier protein through amide bond formation, Michael addiction and reductive amination. Although these spacers efficiently conjugate TACAs and carrier protein together, they also induced immune response against itself. Boon’s group prepared Le^Y^ conjugated KLH vaccine with 4-(maleimidomethyl) cyclohexane-1-carboxylate (MI) linker. The ELISA results indicated strong IgG antibody that recognized linker region was induced [125].

Based on the above results, series of carbohydrate-based anticancer vaccines have been generated and used in clinical trials including gangliosides (GD2, GD3, and GM3), Lewis structure series, O glycans (Tn, STn and Tf) and Globo series (Globo H and SSEA4) [28, 126–131].

#### Polyvalent vaccine

**Fig. 12:**
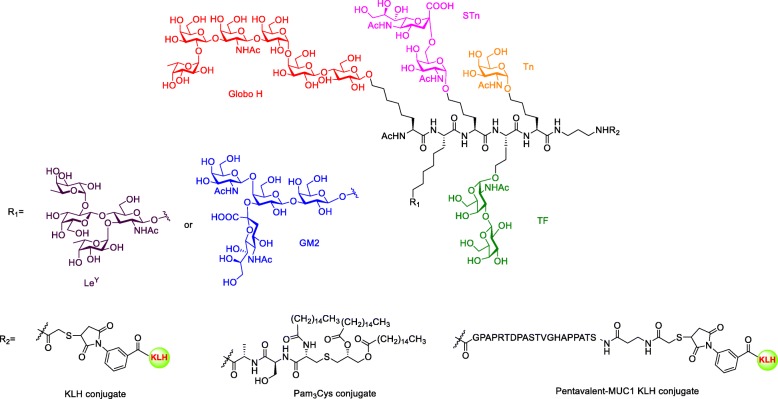
Unimolecular pentavalent vaccine containing Globo H, STn, Tn, LeY or GM2 and TF

With the successful experience in monovalent vaccine development, Danishefsky and Livingston group developed multiple antigens in a one single TACA vaccine. In their Phase II clinical trial, the patients were co-administrated with GM2, Globo H, Lewis^y^, TF(c), Tn(c), STn(c) Tn-MUC1 that was individually conjugated to KLH and mixed with adjuvant QS21 as a heptavalent vaccine. Eight of nine patients developed responses against at least three antigens. However, the antibodies titer was lower than the response from administration of a single corresponding vaccine [132]. The over-dose carrier protein KLH may induce a strong immune response against itself and impair the response against carbohydrate antigens. To overcome this issue, Danishefsky and coworkers first synthesized unimolecular pentavalent vaccine containing Globo-H, STn, Tn, TF, and Le^y^ antigens, which are overexpressed on prostate and breast cancer cell surfaces (Fig. 12) [133]. Then, they attached these antigens to an amino acid by peptide coupling and conjugated the assembly to KLH by Michael addition. The immunological studies of these glycoconjugates showed that antibodies against Globo-H, STn, Tn, and TF were strongly induced in comparison to the pooled monovalent vaccine in the preclinical result. But antibodies against Le^y^ were not as strong, possible due to immune-tolerance caused by relatively high Le^y^ on normal cells. To improve the vaccine efficacy, the same research group developed a second-generation unimolecular pentavalent vaccine, which targets the Globo H, STn, Tn, TF, and GM2 instead of Le^y^ (Fig. 12). The GM2 was selected because the GM2 induced antibodies are able to recognize cancer cell and positively correlated with patient survival in clinical trial [120]. The vaccine induced perspective antibodies not only target each antigen but also recognize the overexpressed antigens on cancer cells [134]. The results of the phase I study of this unimolecular pentavalent vaccine demonstrated vaccine safety and effective induction of antibody responses against five ovarian cancer cell surface antigens. Specifically, IgG and/or IgM titers were detected against 3 or more antigens in 9 out of 12 patients, 4 or more antigens in 7 out of 12 patients, and 5 or more antigens in 3 out of 12 patients [135]. In short, the unimolecular pentavalent vaccines that combined several carbohydrate antigens and carrier protein conjugates could simulate immune response against the heterogeneous carbohydrate epitopes expressed on the surface of cancer cells. In comparison with combined monomeric vaccines, the unimolecular pentavalent vaccine allows higher yield of the final conjugation step, simplified carbohydrate ratio validation step, mimicking the heterogeneity of cancer cells and lower carrier protein amount to minimize the immune suppression.

#### Fully synthetic carrier vaccine

Despite many encouraging preclinical results, many limitations have prevented the carbohydrate-protein conjugate vaccines from FDA approved. First, the yield of the conjugation step is low, and the conjugation numbers are not consistent in each batch, affecting vaccine efficacy. Second, both the carrier protein and the linker between carbohydrate and carrier protein, may also be immunogenic and induce immune response against itself [125]. The undesired antibody production that targets carrier protein and linker may affect the vaccine efficacy and decrease the desired antibody titer. Lee et al. installed the phenyl NO_2_ at the reducing end of glycan and conjugated it to CRM_197_ [136]. After immunization, the glycan array result showed that antiserum from the immunized mice recognized the phenylNO_2_ but not the glycan. This result indicated that the strong immunogenic function group reduces the vaccine efficacy. Yin et al. synthesized Qβ-Tn through click reaction with triazole function group [137]. After immunization, the antiserum bound to the triazole structure and can not recognize the TA3Ha cancer cells. They replaced the triazole to the less immunogenic alkyl amide linker on the Qβ-Tn which was immunized in mice. The antiserum not only bound to the Tn antigen but also recognized the cancer cells. The results indicated that the immunogenic function group at linker moiety result in reduction of vaccine efficacy. To achieve the significance of clinical trial for TACAs vaccine, the strong immunogenic function group like triazole should be avoided. The less immunogenic alkyl amide may be a proper linker for covalent conjugation of TACAs to carrier protein.

**Fig. 13:**
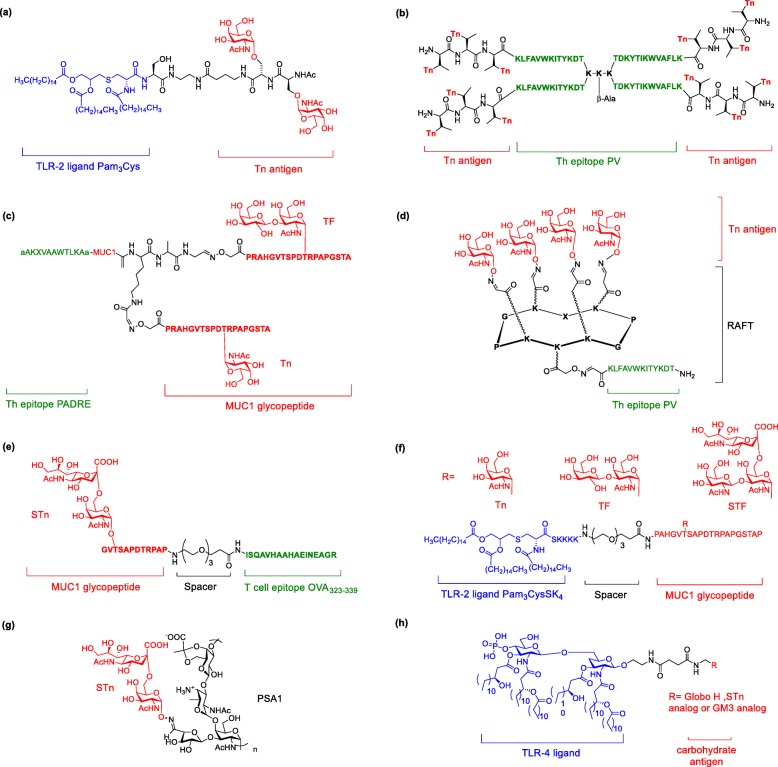
Fully synthetic vaccines. (**a**) Pam3Cys conjugated Tn; (**b**) Th epitope PV conjugated Tn glycopeptides; (**c**) Th epitope PADRE conjugated Tn and Tf-MUC1 glycopeptides; (**d**) Th epitope PV conjugated with RAFT cyclic peptide and tetravalent of Tn; (**e**) T cell epitope OVA conjugated STn-MUC1 glycopeptide; (**f**) Pam3CysSK4 conjugated Tn, Tf or STf-MUC1 glycopeptides; (**g**) PSA1 conjugated STn; (**h**) MPLA conjugated Globo H, STn or GM3

To overcome the disadvantage brought by the carrier protein, many studies attempted to use different epitopes of immune cells to elicit immune response. Agonist of toll-like receptor (TLR) on dendritic cells activates NFkB and AP-1, resulting in cytokine secretion and immune activation. Moreover, Toyokuni et al. were the first to couple Tn antigen to a TLR agonist tripalmitoyl-S-glycerylcysteinylserine (Pam3Cys) as fully synthetic vaccine (Fig. 13a) [138]. Although only moderate IgG was induced, it was the first carrier protein-free TACA vaccine that could elicit immune responses against carbohydrate antigen. To induce IgG antibody production and longterm memory B cells, the involvement of T cell is necessary for antibody affinity maturation in B cells. Cantacuzene’s group synthesized Tn glycopeptide that contains PV as T cell epitope (Fig. 13b). The resulting vaccine induced robust IgG antibodies, which recognized cancer cell line and also increased the survival rate of tumor-bearing mice [139–141]. Another Th cell epitope, Pan DR epitope (PADRE) installed on TACAs was also able to induce robust IgG antibodies titer (Fig. 13c) [142, 143]. Dumy and coworkers designed clustered Tn antigen conjugated on PV regioselectively using addressable functionalized templates (RAFTs). The RAFT glycoconjugates scaffold is a non-immunogenic, built-in vaccine carrier and elicits IgG antibodies that recognize Tn antigens (Fig. 13d) [144]. Kunz’s group connected STn glycopeptides to a Th-cell peptide epitope from ovalbumin (OVA_323–339_) by a non-immunogenic amino acid spacer (Fig. 13e) [145]. The resulting vaccine induced strong and specific immune response against the tumor-associated structure. Later, the same group installed Tn, STn and TF antigens on Pam_3_CysSK_4_ through fragment condensation (Fig. 13f) [146]. Although antiserum titers were not as high as MUC1 tetanus toxoid vaccine, the antibodies only recognized the MUC1 glycopeptides with the same glycosylated site. On the other hand, to avoid the enzymatic degradation and increase the bioavailability of vaccine, BenMohamed *et.al* conjugated Tn mimetics instead of native Tn on RAFT with an immunostimulant peptide epitope (OvaPADRE). This vaccine induced long-lasting and strong IgG/IgM antibodies, which protects mice against tumor progression [147].

Zwitterionic polysaccharides (ZPSs) can induce MHCII mediated immune response and replace carrier protein as a potential component of carbohydrate-based vaccine. De Silva et al. modified PS-A1 to Tn antigen by oxime formation to afford fully carbohydrate vaccine without other immune stimulant [148]. The immunization of this vaccine evoked high titer and specific antibodies. The same group conjugated STn on PS-A1 and characterized the loading amount of STn 1 to be around 10–11% by H NMR integration and Svennerholm method (Fig. 13g) [149]. The immunization of the vaccine with adjuvant elicited strong immune response and high titer IgM/IgG antibodies. These antibodies not only recognized cancer cells (MCF-7 and OVCAR-5) but also conducted complement-dependent cellular cytotoxicity cell lines. Another full carbohydrate vaccine was developed by Guo’s group. They individually conjugated modified GM3, STn, or Globo H on monophosphoryl lipid A (MPLA) to form three built-in adjuvants (Fig. 13h). Among them, Globo H-MPLA vaccines elicited stronger antibodies titer and higher cell toxicity activity without external adjuvant in comparison to Globo H-KLH with Freund’s complete adjuvant [150–153].

**Fig. 14:**
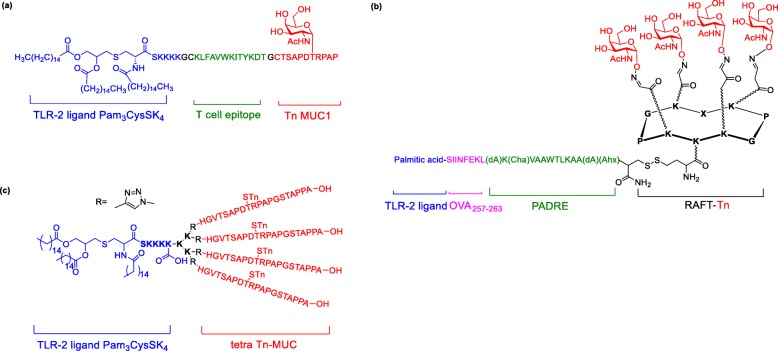
Fully synthetic multicomponent and multivalent vaccines (**a**) Three components vaccine contains Pam3CysSK4 adjuvant, Th epitope and Tn-MUC1; (**b**) Four components vaccine contains palmitic acid adjuvant, OVA CD8+ T cell epitope, PADRE CD4+ T cell epitope and Tn-RAFT; (**c**) Pam3CysSK4 with tetra Tn MUC1 glycopeptides

The above result showed that three components, including B cell epitopes (TACAs), TLR agonist (built in adjuvant) and Th epitope (MHCII presenting peptides), play a crucial role for the fully synthetic vaccine to induce strong, specific and long-lasting immune response. Ingale *et.al* synthesized three components to form fully synthetic vaccine composed of TLR ligand (Pam_3_CysSK_4_), Th epitope (PV) and B epitope (Tn glycopeptide) (Fig. 14a) [154]. The lipid moiety facilitates the uptake of the vaccine by macrophages and dendritic cells. Impressively, the vaccine induced strong antibodies, which were able to recognize cancer cell line even without coadministration of QS-21 adjuvant. Moreover, Th epitopes induced very low antibodies, indicating that immunosuppression was tolerable. Dumy and BenMohamed’s group developed a tetra-components vaccine by assembling a cluster of B cell epitope (Tn antigen), CD4^+^ T cell epitope (Pan-DR), CD8^+^ T cell epitope (OVA_257–264_) and built-in adjuvant (palmitic acid) through oxime and disulfide bond formation (Fig. 14b) [155]. The vaccine significantly induced strong antibodies that recognized tumor cell lines, activated CD4^+^ and CD8^+^ cells, and protected mice against lethal carcinoma cell challenge [156]. Cai et al installed different numbers of Tn or STn glycopeptides into two component vaccine by the click reaction (Fig. 14c). The immunological study result indicated that four copies of a MUC1 sialyl-Tn antigen showed excellent antibodies titer and elicited an antiserum that killed the cancer cells by CDC [157].

#### Modification of TACAs

Although TACAs are generally ideal vaccine candidates, some of them are expressed in normal tissue or cells in the developing stage, leading to immune tolerance and lower immunogenicity of the vaccine. Two types of modified TACA vaccines have been studied including metabolic oligosaccharides engineering (MOE) vaccine and cross-reactivity antibodies induced by modified TACAs. The modification of TACAs vaccine provides the following advantages, 1) preventing the immune tolerance, 2) avoiding the glycosidase degradation, and 3) enhancing the immunogenicity.

#### Metabolic oligosaccharides engineering (MOE)

In this strategy, modified TACA analogs vaccine was immunized to tumor-bearing mice. Then, mice were treated with the corresponding precursor, which was processed into modified TACA on the surface of cancer cells. The antibodies induced by modified TACA analog vaccine were able to recognize the bio-synthesized antigen on the cancer cell and eliminated cancer cells by ADCC or CDC.

Moreover, Guo’s group modified the *N*-acetyl group on the sialic acid of GM3 into different functional groups and conjugated them on KLH [158]. Among them, *N*-phenylacetyl GM3-KLH showed best immunogenicity and T cell-dependent immunity. However, its antisera showed low cross-reactivity in binding to native GM3. They further incubated the cancer cells with corresponding mannosamine and analyzed those cells by FACS [159]. Particularly, *N*-phenylacetyl-D-mannosamine was used as a precursor and synthesized into *N*-phenylacetyl GM3. The modified GM3 expressing cancer cells could go through anti-GM3PAc immune serum-mediated cytotoxicity. Later, they performed both in vitro and in vivo model for *N*-phenylacetyl GM3 expression. The mice treated with *N*-phenylacetyl mannosamine showed strong *N*-phenylacetyl GM3 expression. The *N*-phenylacetyl GM3 vaccine protected mice against tumor progression after metabolic oligosaccharides engineering. Another TACA STn was also modified into *N*-phenylacety and *N*-chlorophenylacetyl STn by the same group, and the vaccine immunogenicity was also stronger than native STn vaccine [160–162].

These results demonstrated that MOE is a powerful tool to enhance immunogenicity. Most studies have focused on sialic acid modification. However, sialic acid plays many important roles in biological function. Unnatural sialic acid may contribute to breakage of its original function and result in disease. Hence, the investigation of MOE side effects is required in the future.

#### Cross-reactivity antibodies induced by modified TACAs

**Fig. 15:**
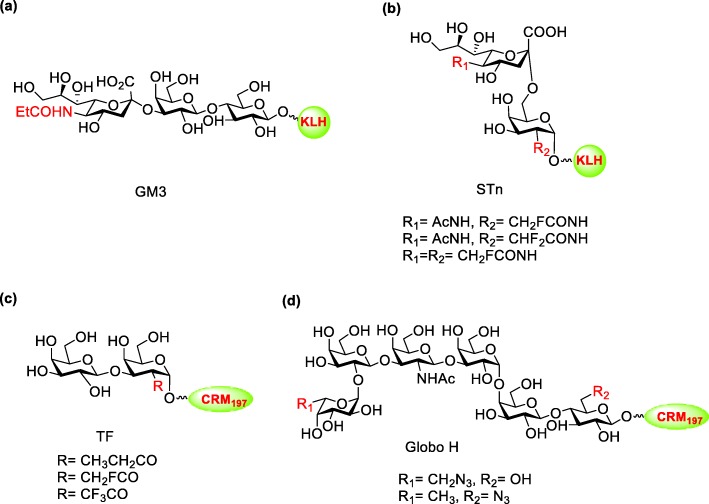
TACA modification vaccines (**a**) propionamide modified GM3-KLH; (**b**) *N*-fluoroacetyl modified STn-KLH; (**c**) *N*-fluoroacetyl modified TF-CRM_197_; (**d**) azido modified Globo H-CRM_197_

To overcome the shortage of MOE, many studies focus on modification of TACAs vaccines, which not only could generate stronger immunogenicity but also induce cross-reactive antibodies recognizing native carbohydrate antigens on the tumor cells. Zheng et al. synthesized a series of GM3 analogs with the modification at *N*-acetyl group on sialic acid (Fig. 15a) [163]. The GM3-KLH vaccine with propionamide elicited higher IgM and IgG titer than native GM3 vaccine. Besides, those antibodies are highly cross-reactive to native GM3, indicating that modification of TACA can generate not only stronger immunogenicity but also cross-reactivity to native antigen.

STn antigen has also been modified and studied in many studies. Ye’ s group reported different modifications at N-acetyl group on the sialic acid of STn [164]. The vaccines with fluorine modified STn showed stronger IgG titer and higher IgG/IgM ratio in comparison to native STn vaccine (Fig. 15b). To enhanced the vaccine stability and avoid the glycosidase hydrolysis, they also substituted the oxygen at the glycosidic bond to sulfur to generate S-linked STn derivatives with fluorine-containing modification [165]. Even though the vaccines could elicit cross-reactive antibodies to recognize native STn, the antibodies titer was not stronger than native STn vaccine. In vivo result indicated that ***N*-**fluoroacetyl modified STn vaccine was able to induce T cell-dependent immunity, increase survival in tumor-bearing mice and activate antibodies mediated cell cytotoxicity (ADCC and CDC) [166]. Similar modifications were installed at N- acetyl group at TF antigen (Fig. 15c) [167]. Compare to native TF vaccine, N-fluoroacetyl modified TF vaccine induced two-fold IgG antibodies titer. Although some modified vaccines showed remarkable results, and most of them targeted an amino group, which can be selectively converted to other function groups instead of majority hydroxyl on the carbohydrates. The specific modification at the hydroxyl group is more challenging because complicated protection and deprotection procedures are required for the installation of site-specific modification in numerous hydroxyl group. Our group used chemoenzymatic **s**trategy to synthesize numerous Globo H analog vaccines with the modification at reducing and non-reducing end [136]. Our results indicated that azido modification at the nonreducing end of Globo H-CRM_197_ could elicit stronger IgG titer than native Globo H vaccine (Fig. 15d). The antiserum was able to recognize the cancer cell line and eliminate it by ADCC.

## Future prospective and conclusions

Generally speaking, prevention is better than treatment, and vaccination is an effective and safe approach to prevent infections. Since the last century most of the diseases such as polio, smallpox, rubella, influenza, mumps and other have been under controlled, and some diseases now are even completely eradicated after the introduction of traditional vaccines (live and killed vaccines) [168].

Moreover, the glycoconjugate vaccines such as *S. pneumoniae*, *H. influenzae* and *N. Meningitidis,* which are made by poor immunogenic oligo−/polysaccharide covalently linked to carrier protein (T-cell epitope), exhibit high efficiency and effectively worked for chilfren younger than 2 years of age. Unfortunately, these vaccines are not readily available for children in poor countries due to their high cost and low supply. Also, these glycoconjugate vaccines are able to protect people from vaccinated serotypes, but recently reported emergency of non-vaccine serotypes of *S. pneumoniae* and *N. Meningitidis*. Therefore, more studies on serotype inclusion or replacement are needed.

Although conjugate vaccines are effective and safe, but some issues need to be addressed. There is no general rule to predict the optimal length/size of the oligosaccharide and appropriate saccharide/protein molar ratio for vaccine development. Moreover, the presence of carrier protein and linker in conjugate vaccines can lead to some disadvantages. Both carrier proteins and linkers themselves can be immunogenic and elicit nonspecific immune response that can suppress carbohydrate-specific antibody production [169]. Therefore, there is a need to design and develop carrier protein free and linker free vaccines. The recent studied zwitterionic polysaccharide (ZPS) type vaccines are an alternative. The ZPS vaccines contain both positive and negative charges on adjacent monosaccharide units and were found to be able to elicit MHC II mediated T cell response without linkage of carrier protein [170]. This finding has important implications for the design of novel polysaccharide vaccines.

The development of carbohydrate-based anti-cancer vaccine has made significant progress in the past few decades. Preclinical trials of monovalent and polyvalent vaccines showed encouraging results. With more understanding about the carrier protein, many fully synthetic carbohydrate vaccines with good immunogenicity, low linker effect and optimized conjugation step between carbohydrate and immune-stimulant moiety have being developed. However, there is still a major gap between the mice models and clinical trials. So far, no TACAs vaccine has been approved by the FDA. The slight expression of TACAs on normal tissue may result in the immune-tolerance and lead to low immunogenicity in clinical trial. Although a proper model to determine the immunogenicity in human remains to be developed, modification of TACAs to generate “non-self” antigen vaccine and induce cross-reactive antibody will be a good tool for the future studies.

Overall, with the experiences in vaccine development and clinical trials, carbohydrate-based anti-cancer vaccine seems to be closer to reach than ever. More efforts are still needed to deal with low immunogenicity issue, unsound immune system in patients, TACSs expression level between cancer and normal cells in patients, and the protocol design for clinical trials.

## Data Availability

Not applicable.

## Abbreviations

BSA: Bovine serum albumin
CRM_197_: Non-toxic mutant of diphtheria toxin
DT: Diphtheria toxoid
ELISA: Enzyme linked immunosorbent assay
HSA: Human serum albumin
KLH: Keyhole limpet hemocyanin
OMP: Outer membrane vesicle
OVA: Ovalbumin
TT: Tetanus toxoid
